# A Wearable High-Resolution Facial Electromyography for Long Term Recordings in Freely Behaving Humans

**DOI:** 10.1038/s41598-018-20567-y

**Published:** 2018-02-01

**Authors:** Lilah Inzelberg, David Rand, Stanislav Steinberg, Moshe David-Pur, Yael Hanein

**Affiliations:** 1 Tel Aviv University Center for Nanoscience and Nanotechnology, Tel Aviv University, Tel Aviv, Israel; 2 School of Electrical Engineering, Tel Aviv University, Tel Aviv, Israel; 3 Sagol School of Neuroscience, Tel Aviv University, Tel Aviv, Israel

## Abstract

Human facial expressions are a complex capacity, carrying important psychological and neurological information. Facial expressions typically involve the co-activation of several muscles; they vary between individuals, between voluntary versus spontaneous expressions, and depend strongly on personal interpretation. Accordingly, while high-resolution recording of muscle activation in a non-laboratory setting offers exciting opportunities, it remains a major challenge. This paper describes a wearable and non-invasive method for objective mapping of facial muscle activation and demonstrates its application in a natural setting. We focus on muscle activation associated with “enjoyment”, “social” and “masked” smiles; three categories with distinct social meanings. We use an innovative, dry, soft electrode array designed specifically for facial surface electromyography recording, a customized independent component analysis algorithm, and a short training procedure to achieve the desired mapping. First, identification of the *orbicularis oculi* and the *levator labii superioris* was demonstrated from voluntary expressions. Second, the *zygomaticus major* was identified from voluntary and spontaneous Duchenne and non-Duchenne smiles. Finally, using a wireless device in an unmodified work environment revealed expressions of diverse emotions in face-to-face interaction. Our high-resolution and crosstalk-free mapping, along with excellent user-convenience, opens new opportunities in gaming, virtual-reality, bio-feedback and objective psychological and neurological assessment.

## Introduction

Human facial expressions fascinate and elude scientists despite decades of extensive investigations^1–3^. New insights continue to emerge, supporting universality on one hand, with astounding complexity on the other hand^4^. Smiling, as a special case, is among the most complex facial expressions, involving no fewer than 7 different unilateral muscles^5,6^. Smiling holds great importance in human development and communication, and it is one of the first expressions to appear in developmental stages. Despite their ubiquitous nature, smiles remain an elusive and debated topic. Commonly associated with happiness, smiles have very diverse meanings including appeasement and greeting. It is widely accepted that smiles of felt joy or enjoyment (true smiles) are distinct from non-felt joy or social smiles (false/fake smiles). A social smile is a phony smile reflecting an attempt to appear positive^7,8^. Both smiles apply the contraction of the *zygomaticus major* muscle (pulls the lip corners up), but the activation of the *orbicularis oculi* muscle (surrounding the eye), is long considered to be a unique hallmark of spontaneous enjoyment^9^. Ekman and Friesen posited that smiles reflecting enjoyment can be identified by special markers such as synchronization between the *zygomaticus major* and the *orbicularis oculi*, symmetrical activation of both *zygomaticus major* muscles, and activation duration^9,10^. Recent investigations demonstrated that Duchanne smile can be produced voluntarily, evidenced by co- activating the *orbicularis oculi* and *zygomaticus major*^11,12^. Challengingly, smiles may mask anger, disgust and other negative emotions. One such masked smile is a sneer expression, representing contempt or disgust (activation of the ‘Nose Wrinkler’, namely the *levator labii superioris)*, concealed by the *zygomaticus major* activation^5,6,13^. Masked smiles have received relatively little attention, yet they may in fact have important diagnostic value^14^.

The unavailability of a reliable and non-interfering experimental tool to characterize facial muscle activation remains an unmet challenge. Most notably, the need to reach single muscle specificity without tampering with the emotional-state of the tested subject has proved difficult to achieve. While visual analysis has gained huge acceptance in recent years^7,9^, and has been enriched by automated facial analysis methods^15^, it does not capture fundamental muscle activation. In fact, many facial movements affect nearby regions and screen the precise muscle source and timing, especially under strong muscle activation conditions. Surface electromyography (sEMG) is an important alternative, owing to its ability to directly detect the electrical activity of muscles^16^.

The main strength of facial sEMG is in its potential ability to provide precise physiological information by identifying specific muscle activation, while also negating the need for a constant visual path to the subject’s face. Yet, sEMG usefulness depends on the ability to overcome crosstalk and to achieve high resolution and specificity^17,18^, along with subject comfort. So far, high-resolution facial sEMG relied on gelled electrodes and lengthy placement procedures, and was therefore restricted to artificial laboratory settings.

In the present work, we demonstrate a powerful new facial sEMG system and show its unique performances in capturing three muscles involved in different smiles. First, novel electrodes were realized to achieve optimized user experience. The electrodes are extremely soft and flexible and have a compact electrical interface, thus minimizing user discomfort or distraction by the measurement setup. Wireless setup further supports the use of the technology in a natural setting. Second, an independent component analysis (ICA) algorithm was adapted to construct muscular activation maps of specific muscles. Third, a simple and short training and validation procedure was developed and applied. Finally, an application of the technology in a completely natural work environment demonstrated the capacity to identify the activation of the *orbicularis oculi*, the *zygomaticus major*, and the *levator labii superioris* muscles in face-to-face interactions.

## Results

**Figure 1:**
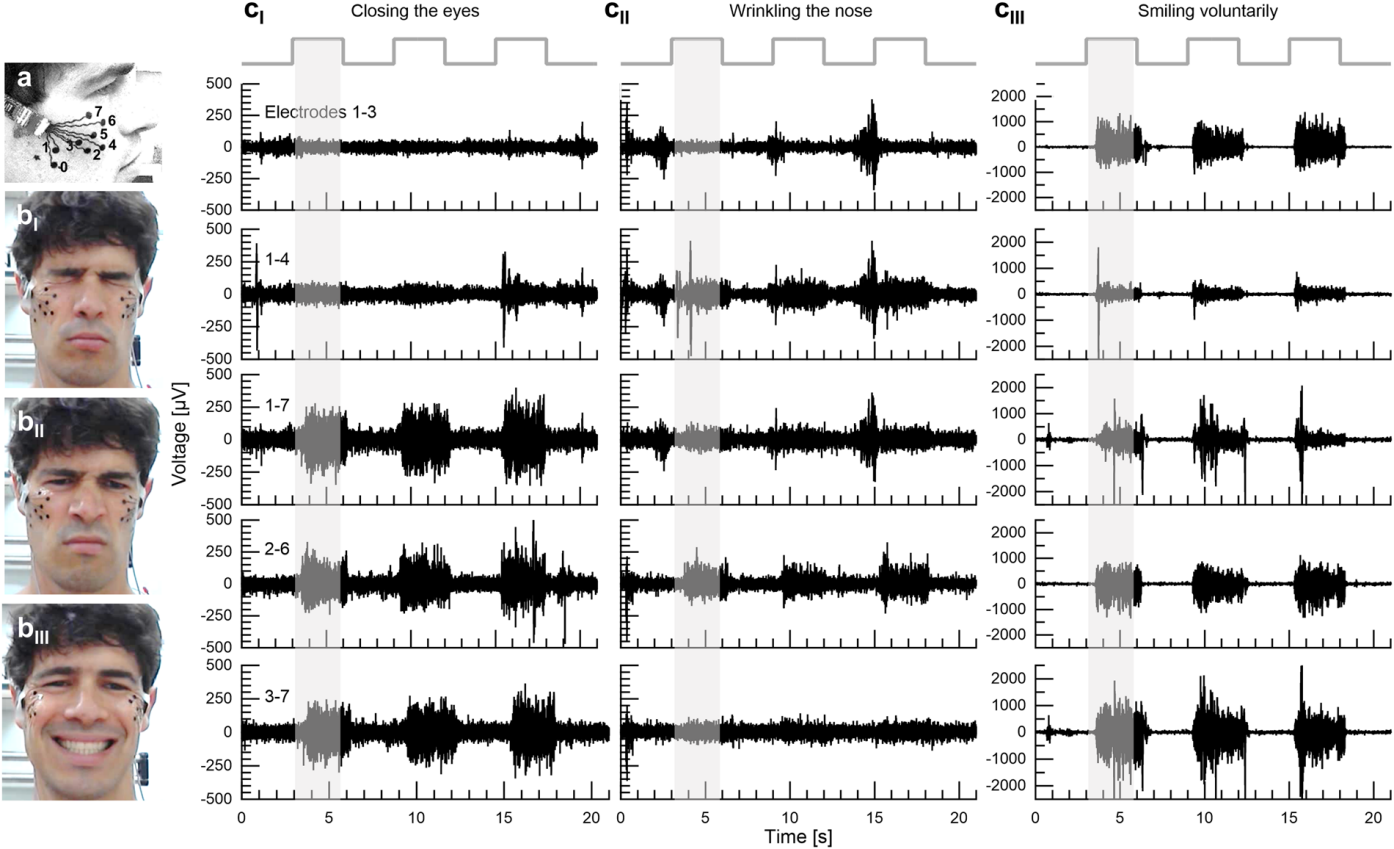
Three facial voluntary activations and their corresponding differential sEMG (filtered) data from 5 electrode pairs (**a**) Electrode array placement (electrodes 0 to 7). (**b**) Three facial tasks: (**b**_I_) Closing the eyes; (**b**_II_) Wrinkling the nose; (**b**_III_) Smiling voluntarily. (**c**) Differential sEMG (voltage versus time) of electrode pairs 1–3, 1–4, 1–7, 2–6 and 3–7 during three repetitions (shaded areas indicate task onset times) of: (**c**_I_) Closing the eyes; (**c**_II_) Nose-wrinkling; (**c**_III_) Smiling voluntarily (expected to activate the *zygomaticus major* muscle region). Smiling is typified by a very large amplitude activation in almost all electrodes (note the difference in voltage scale).

To identify the *orbicularis oculi*, the *zygomaticus major*, and the *levator labii superioris* muscles (the principle muscles in “enjoyment”, “social” and “masked” smiles respectively), we used specifically designed dry electrode arrays as was previously described^19^ (see Methods and Fig. 1a). For convenience, the initial stage of the investigation was performed with a wired system allowing easy triggering and on-line data evaluation. Two electrode arrays were connected to two amplifier units, (Intan Technologies amplifier evaluation board, RHD2000) using a costume-made printed circuit board (PCB) connector. The arrays were adhered to the left and right cheeks of healthy volunteers after a mild skin cleaning and exfoliation. For electrode placement, subjects were asked to smile and close their eyes to locate the *zygomaticus major* (electrodes 2–5) and *orbicularis oculi* (electrodes 6–7) muscles area, respectively. The rough direction of the *zygomaticus major* was identified along the direction of the pulled lips during smiling and that of the *orbicularis oculi* by the contracted region surrounding the eyes (Fig. 1a). The same electrode array design was used with all volunteers and did not account for anatomical facial differences. A commercial ground plate electrode (Natus) was placed at the back of the neck. The placement procedure took about a minute and the recording started immediately after placement and continued for an hour. Overall, sEMG recording was performed on 18 healthy volunteers (age: 31.58 ± 3.41 years, 13 females). Subjects sat in a relaxed upright position and were instructed to imitate photographs of facial expressions presented on a computer monitor (from Schumann *et al*., 2010^20^). The volunteers were allowed to move their head freely during the recording and their facial expressions were simultaneously video-recorded for later evaluation.

The volunteers were first instructed to imitate the following three static facial expressions: closing the eyes forcefully, wrinkling the nose and smiling voluntarily while holding each expression for 3 s with a 3 s gap of calm, neutral expression in between (Fig. 1b_I_–b_III_). Each facial expression was presented 9 times. Photographs were shown in a random order. Single-ended data signal-to-noise ratios (SNR) varied between tasks and individuals. SNR (in all subjects) of wrinkling the nose was 1.57 ± 0.43 (differential *SNR* = 4.66 ± 2.18); of closing the eyes was 1.97 ± 0.65 (differential *SNR* = 10.86 ± 7.44) and of smiling voluntarily was 4.06 ± 2.59 (differential *SNR* = 7.73 ± 4.64). Baseline noise root mean square (RMS) was 39.82 ± 6.58 *μV* for the single-ended data and 13.78 ± 4.90 *μV* for the differential data, over all subjects and electrodes (Supplementary Fig. S1).

Figure 1 shows differential sEMG data from electrode pairs: 1–3, 1–4, 1–7, 2–6 and 3–7 recorded from the same subject. Closing the eyes was recorded in electrodes 6 and 7, at close proximity to the *orbicularis oculi* muscle region. Nose-wrinkling was picked primarily by electrode 6, close to the *levator labii superioris* muscle region. Smiling voluntarily activated the *zygomaticus major* muscle region, in agreement with the results reported by Schumann *et al*. (Fig. 1c_I_–c_III_)^20^ (see also Supplementary Fig. S2). It is important to note the significantly stronger amplitude of the smile task compared to the two other tasks.

While the data in Fig. 1 clearly reveals discrimination between different tasks, associating the recorded signals with specific muscles is not straightforward. Primarily, crosstalk from different muscles, especially those with high activation intensity, such as the *zygomaticus major* during smiling, is readily apparent. Moreover, since electrode layout was fixed, electrode placement relative to specific muscles varied slightly for different subjects. These challenges were overcome using independent component analysis (ICA), a specific blind source separation (BSS) algorithm used in sEMG analysis^21–23^, to extract and validate activation maps.

**Figure 2:**
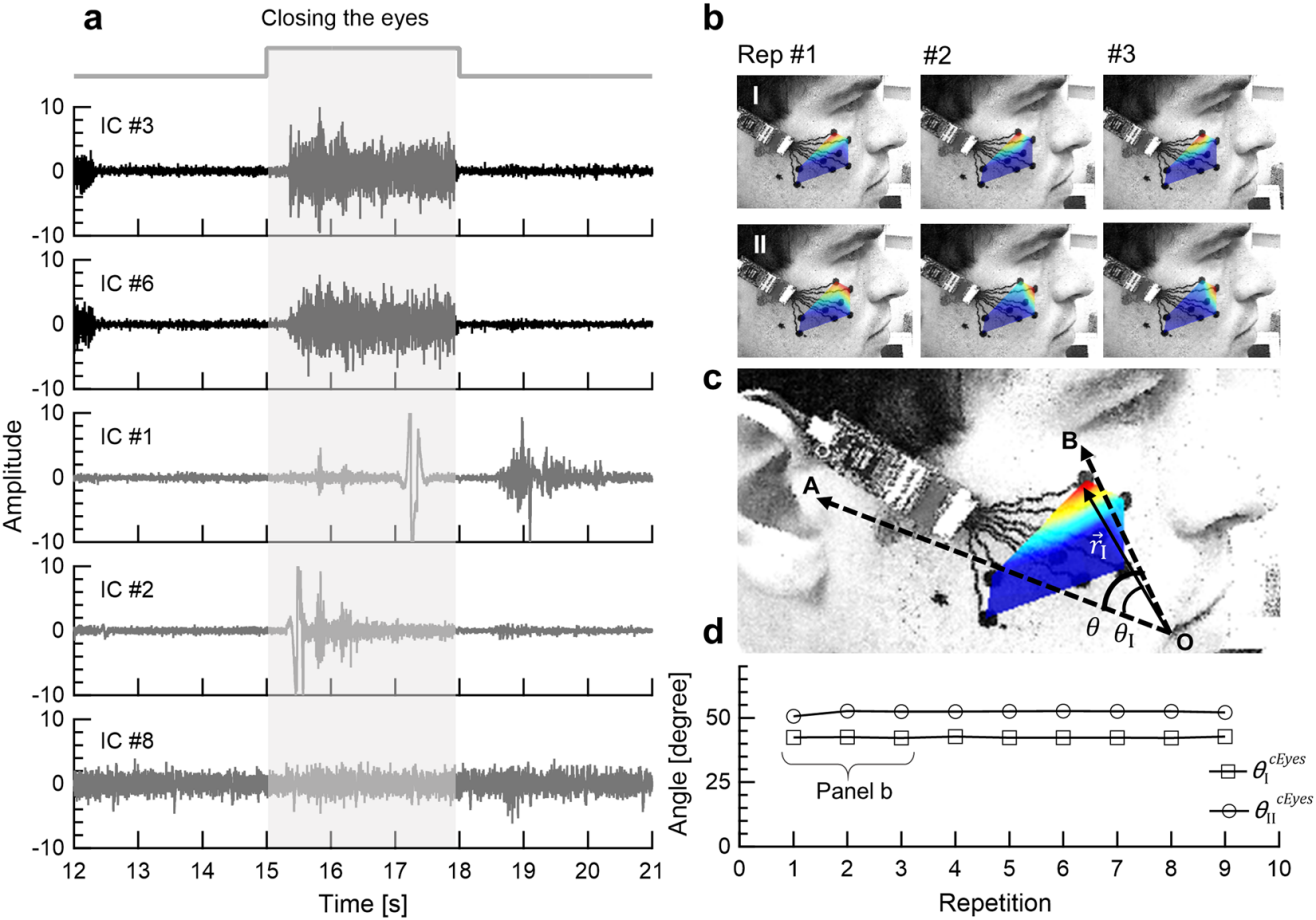
ICA of closing the eyes task. (**a**) IC amplitude versus time (second repetition). ICs #3 and 6 correspond to sEMG signal while the others (ICs #1, 2, 4, 5, 7 and 8) are noise components (Shaded areas indicate task onset time). (**b**) IC maps during three consecutive repetitions of closing the eyes. Red color indicates highest muscle activation. (**c**) Subject geometrical sector was defined by two principle vectors, namely OA and OB; such that O, A and B were located at the subject’s mouth corner, ear tragus and eye corner, respectively. *θ* is defined as the sector angle. (**d**) IC angles (${\theta }_{{\rm{I}}}^{cEyes}$ and ${\theta }_{{\rm{II}}}^{cEyes}$, corresponding to ${{\rm{IC}}}_{{\rm{I}}}^{cEyes}$ and ${{\rm{IC}}}_{{\rm{II}}}^{cEyes}$ respectively) at 9 consecutive repetitions of the closing the eyes task. The first 3 repetitions are shown in (**b**).

We applied the fastICA algorithm using the methodology of Hyvärinen *et al*.^24^. The algorithm output were adapted to facial mapping using the MATLAB fastICA 2.5 package. For each facial task repetition, we applied the fastICA algorithm separately. We calculated the mixing and un-mixing matrices from the original 8 sEMG single-ended data. This process revealed several independent components (ICs, see Fig. 2a), corresponding to sources (depending on task, repetition and individual), and their weight in each electrode. We used electrode location, derived from lateral photographs (during neutral facial expression), to interpolate the absolute values of the inverse unmixing matrix on the image surface. These projections revealed the IC maps for each facial expression in each repetition separately (see Fig. 2b).

Figure 2a shows the amplitude of 5 ICs (out of 8) versus time, derived from 8 single-ended data channels of a single subject (for a single repetition) performing the closing the eyes task. Two ICs have clear sEMG signals (IC #3 and 6), while the others represent noise components. The output of the algorithm yields IC numbers that do not have a consistent meaning from one repetition to the next. This challenge was resolved by grouping IC maps from consecutive repartitions based on visual similarities and later by angle calculations. The interpolated color maps were defined as a JKLMN pentagon, such that the vertex closest to the eye is J. The grouping criteria was based on the location of the red area (maximal muscle activation): closest to J or K (corresponding to the two ICs of closing the eyes task: ${{\rm{IC}}}_{{\rm{I}}}^{cEyes}$ and ${{\rm{IC}}}_{{\rm{II}}}^{cEyes}$).

The IC maps of the first three consecutive repetitions (out of 9) are shown in Fig. 2b (red denotes the highest activation area). As expected, in all repetitions a consistent activation is observed close to the eye (Fig. 2b_I_). A second component is located close to the nose region (Fig. 2b_II_).

We defined for each IC and for each repetition the angles *θ*_*i*_ to facilitate a simple comparison between repetitions (Fig. 2c). *θ*_*i*_ was defined as the angle between ${\overrightarrow{r}}_{i}$ (the vector from the origin, O, to the muscle maximal activation point), and AO (O the origin is at the corner of the mouth, A at the ear tragus and B at the eye corner). In Fig. 2d we plotted ${\theta }_{{\rm{I}}}^{cEyes}$ and ${\theta }_{{\rm{II}}}^{cEyes}$ (corresponding to ${{\rm{IC}}}_{{\rm{I}}}^{cEyes}$ and ${{\rm{IC}}}_{{\rm{II}}}^{cEyes}$, respectively) for 9 consecutive repetitions of the closing the eyes task. The first IC of the closing-the-eyes task, ${{\rm{IC}}}_{{\rm{I}}}^{cEyes}$, represents a consistent source that can be readily associated with the *orbicularis oculi* muscle at ${\theta }_{{\rm{I}}}^{cEyes}=42.40^\circ \pm 0.22^\circ$ The second component, ${{\rm{IC}}}_{{\rm{II}}}^{cEyes}$, stabilized after the second activation (Fig. 2d) at ${\theta }_{{\rm{II}}}^{cEyes}=52.49^\circ \pm 0.19^\circ$ (excluding the outlying first repetition by Grubb’s test).

**Figure 3:**
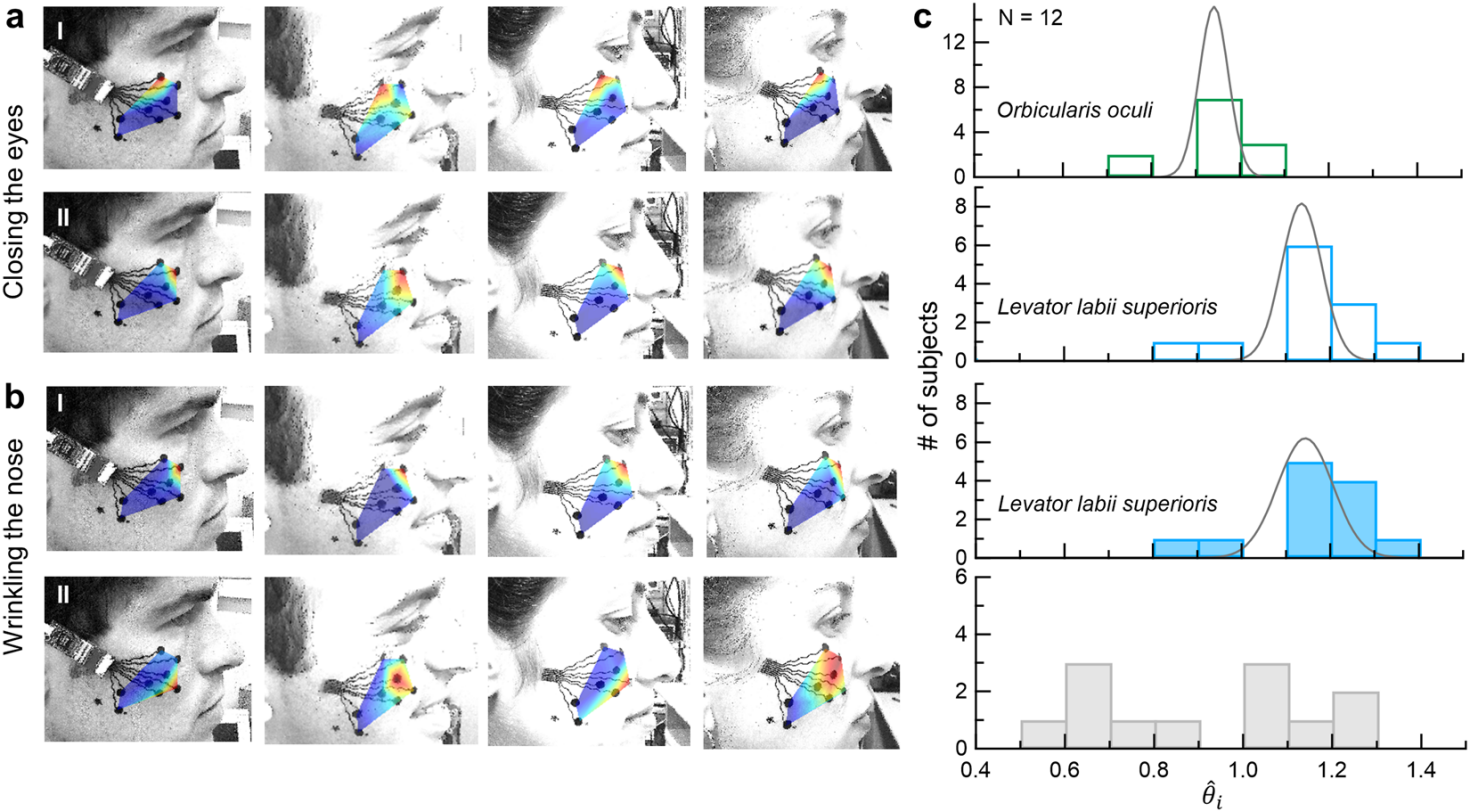
ICA comparison between different individuals. (**a**,**b**) IC maps for closing the eyes and nose-wrinkling tasks. Red color indicates highest muscle activation. Top and bottom rows in (**a**) and top row in (**b**) were organized according to visual similarities. (**c**) Histograms of relative activation angles (${\hat{\theta }}_{i}={\theta }_{i}/\theta )\,\,$for the two tasks. In all subjects ${{\rm{IC}}}_{{\rm{I}}}^{cEyes}$ and ${\,\mathrm{IC}}_{{\rm{II}}}^{cEyes}$ is associated with the *orbicularis oculi* and the *levator labii suprioris* muscles, respectively (first and second rows). In the nose-wrinkling task, ${{\rm{IC}}}_{{\rm{I}}}^{wNose}$ is the *levator labii suprioris* while ${{\rm{IC}}}_{{\rm{II}}}^{wNose}$ varied among subjects (third and fourth rows, respectively).

The robustness of the primary *orbicularis oculi* component, ${{\rm{IC}}}_{{\rm{I}}}^{cEyes}$, over consecutive repetitions was consistent between different subjects (Fig. 3a-top). The first IC map for the nose-wrinkling task, ${{\rm{IC}}}_{{\rm{I}}}^{wNose}$, was also consistent among subjects (Fig. 3b-top). The consistent activation, ${{\rm{IC}}}_{{\rm{I}}}^{wNose}$, (over repetitions and individuals) can therefore be associated with the *levator labii superioris* muscle. It is important to note the similarity between ${{\rm{IC}}}_{{\rm{I}}}^{wNose}$ (Fig. 3b-top) and ${{\rm{IC}}}_{{\rm{II}}}^{cEyes}$ (Fig. 3a-bottom and 2b_II_).

To compare IC locations of different subjects, we calculated the relative angles, ${\hat{\theta }}_{i}={\theta }_{i}/\theta$. *θ* for each subject is defined as the angle between OA and OB (Fig. 2c). Histograms of the relative angles, ${\hat{\theta }}_{i}$, of ${{\rm{IC}}}_{{\rm{I}}}^{cEyes}$, ${{\rm{IC}}}_{{\rm{II}}}^{cEyes}$, and ${{\rm{IC}}}_{{\rm{I}}}^{wNose}$ show consistent activation between subjects and also similarity between ${{\rm{IC}}}_{{\rm{II}}}^{cEyes}$ and ${{\rm{IC}}}_{{\rm{I}}}^{wNose}$. The relative angles of ${{\rm{IC}}}_{{\rm{I}}}^{cEyes}$ and ${{\rm{IC}}}_{{\rm{II}}}^{cEyes}$ were ${\hat{\theta }}_{{\rm{I}}}^{cEyes}=0.94\pm 0.09$ (95% confidence interval of the mean (95%CI) = [0.88–1.00]) and ${\hat{\theta }}_{{\rm{II}}}^{cEyes}=1.14\pm 0.12$ (95%CI = [1.06–1.21]; Fig. 3a-bottom), respectively. Linear mixed effect regression predicting ${\hat{\theta }}_{i}$ was performed to test the random Subject effect and fixed IC effect. The effect of IC was highly significant (${\chi }^{2}(1)=284.36,p < 1.1{e}^{-15}$), indicating that ${\hat{\theta }}_{{\rm{I}}}^{cEyes}$ and ${\hat{\theta }}_{{\rm{II}}}^{cEyes}$ are two separable angles corresponding to two different sources. The relative angle, ${\hat{\theta }}_{i}$, of ${{\rm{IC}}}_{{\rm{I}}}^{wNose}$ was ${\hat{\theta }}_{{\rm{I}}}^{wNose}=1.15\pm 0.12$ (95%CI = [1.07–1.23]; Fig. 3b-top). Another linear mixed effect regression model was performed to predict ${\hat{\theta }}_{i}$ with an additional fixed Task effect (closing the eyes/wrinkling the nose) and the interaction of IC and Task. Task did not influence the relative angle, ${\hat{\theta }}_{i}$ (${\chi }^{2}(1)=0.0023,p=0.962$). Moreover, the IC effect did not benefit from the interaction of IC and Task (${\chi }^{2}(1)=0.0188,p=0.8909$). Thus, we conclude that the relative angles ${\hat{\theta }}_{{\rm{II}}}^{cEyes}$ and ${\hat{\theta }}_{{\rm{I}}}^{wNose}$ are indistinguishable. Therefore, we identify the activation of the *levator labii superioris* as the second component in the closing the eyes task. In summary, for all subjects, the fastICA algorithm revealed at least two components, in the closing the eyes task, which we associate with the *orbicularis oculi* and *levator labii superioris* muscles (Fig. 3a,c top two panels).

**Figure 4:**
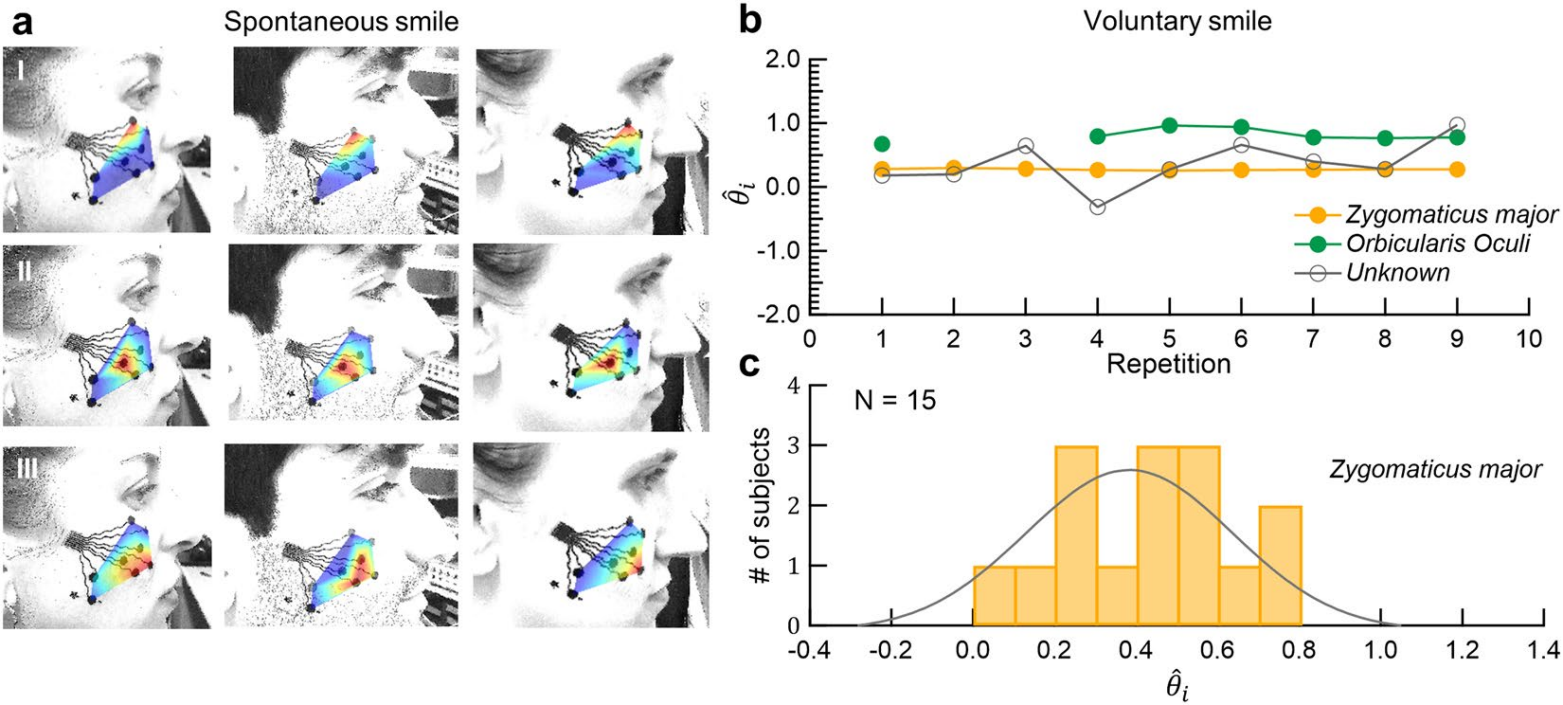
Spontaneous and voluntary smiling. (**a**) IC maps. Red color indicates highest muscle activation. Spontaneous smiling shows clear *orbicularis oculi* (${{\rm{IC}}}_{{\rm{I}}}^{sSmile}$, top) *and zygomaticus major* (${{\rm{IC}}}_{{\rm{II}}}^{sSmile}$, middle) activations along with additional, subject specific (unknown) muscle activations (${{\rm{IC}}}_{{\rm{III}}}^{sSmile}$, bottom). (**b**) Relative angles $({\hat{\theta }}_{i}={\theta }_{i}/\theta )$ in 9 consecutive repetitions of the *zygomaticus major* and *orbicularis oculi* muscles in a single subject. Additional ICs (unknown) are activated, varying along the task. (**c**) ${\hat{\theta }}_{i}$ histogram of the *zygomaticus major* muscle for 15 subjects in the smiling voluntarily task.

Closing the eyes and wrinkling the nose are relatively simple tasks in the sense that they: (1) have a clear primary muscle activation (the *orbicularis oculi* and the *levator labii superioris*, respectively), and (2) activate few ICs (2–4 muscles). Both voluntary and spontaneous smiling is associated with a large number of activated muscles and inter-subject variability. Voluntary smiles in particular varied dramatically between subjects, showing 3 to 6 ICs. To identify the primary smiling muscle, *zygomaticus major*, we used facial sEMG data recorded when volunteers watched a funny video (a skateboarding cat^25^) that evokes spontaneous positive responses. The volunteers were instructed to watch the movie while avoiding talking. Each recording begun with a synchronizing trigger between the sEMG recordings and the video. Although video stimuli were not validated or standardized, all volunteers presented positive facial emotional expressions ranging from smiling to bursts of laughter at least two well-identified time frames. IC of the data of these time episodes (duration of 1–3 s) revealed a clear activation of the *orbicularis oculi*, ${{\rm{IC}}}_{{\rm{I}}}^{sSmile}$ (primary IC of the spontaneous smile), as identified from the closing the eyes task, ${{\rm{IC}}}_{{\rm{I}}}^{cEyes}$: ${\hat{\theta }}_{{\rm{I}}}^{sSmile}=0.88\pm 0.20$ (Fig. 4a-top). Linear mixed effect regression predicting ${\hat{\theta }}_{i}$, of a random Subject effect and a fixed IC effect did not benefit from the interaction of IC and Task (smiling voluntarily/closing the eyes) (${\chi }^{2}(1)=0.11,p=0.74$), indicating that ${\hat{\theta }}_{{\rm{I}}}^{sSmile}$ and ${\hat{\theta }}_{{\rm{I}}}^{cEyes}$ are the same. A second robust component,$\,{{\rm{IC}}}_{{\rm{II}}}^{sSmile}$_,_ we associate with the *zygomaticus major* muscle (${\hat{\theta }}_{{\rm{II}}}^{sSmile}=0.43\pm 0.22$) (Fig. 4a-middle). All subjects had 1 to 4 additional ICs. We used the relative angle, ${\hat{\theta }}_{i}$, to follow these principle ICs over 9 repetitions during voluntary smiles (Fig. 4b). To verify that ${\hat{\theta }}_{{\rm{I}}}^{vSmile}$ and ${\hat{\theta }}_{{\rm{II}}}^{vSmile}$ are two separable angles corresponding to two different muscles we performed an additional regression model with a random Subject effect and a fixed IC effect. Indeed, the IC effect was highly significant (${\chi }^{2}(1)=253.94,p < 1.1{e}^{-15}$). Voluntary smiling yielded muscle activation patterns similar to the one exhibited in spontaneous smiles. It is interesting to note that some subjects activated the *orbicularis oculi* muscle together with the *zygomaticus major* muscle during voluntary smiling, in agreement with previous reports^11,12^. The histogram of the relative angle, ${\hat{\theta }}_{i}$, of the *zygomaticus major* muscle over 15 subjects is shown in Fig. 4c (${\hat{\theta }}_{{\rm{II}}}^{vSmile}=0.43\pm 0.21$, 95%CI = [0.32–0.55]). Compared with the *orbicularis oculi* and *levator labii superioris* histograms (Fig. 3c), the *zygomaticus major* distribution is scattered (Fig. 4c).

**Figure 5:**
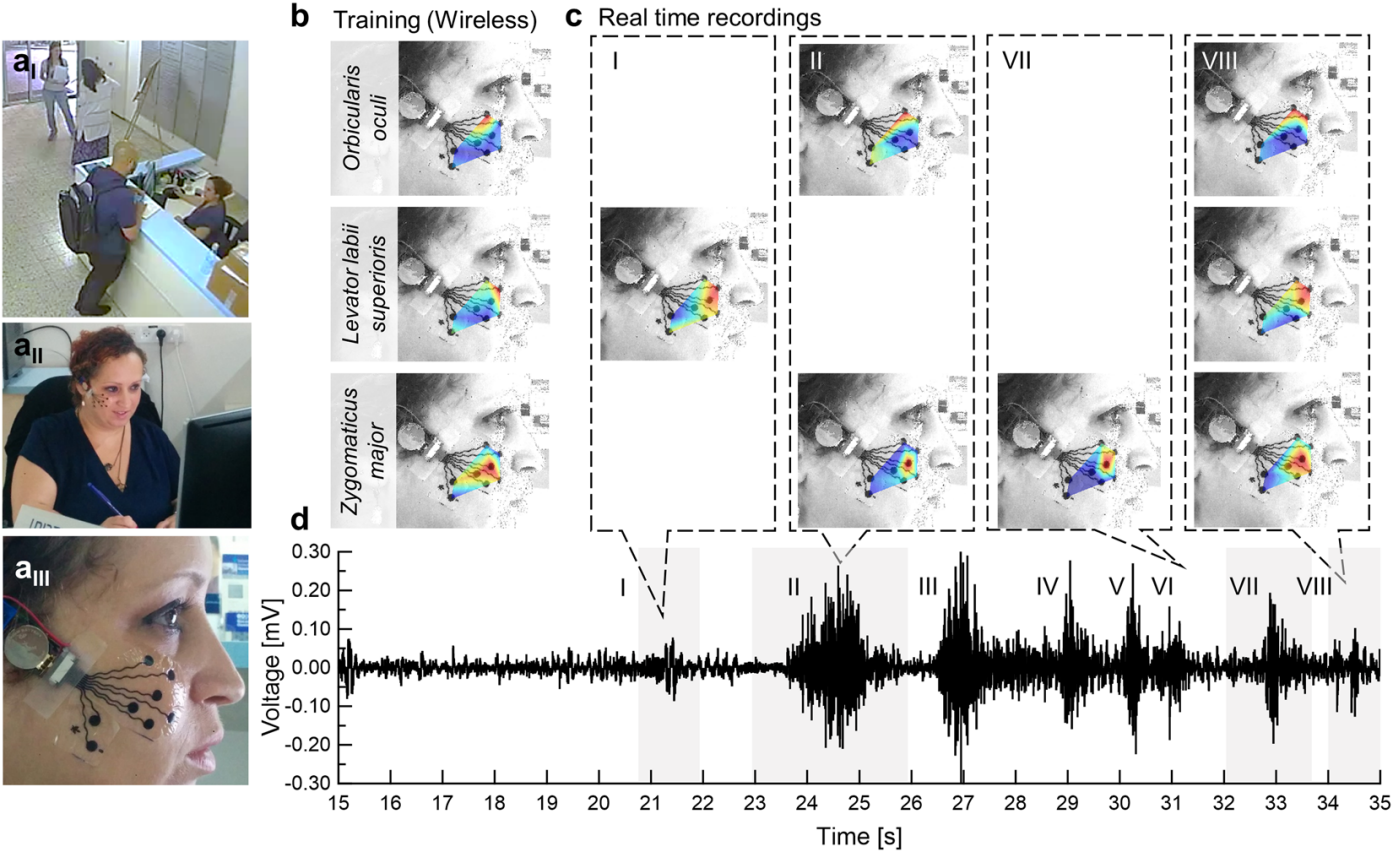
Wireless facial sEMG recordings in a work environment. (**a**_I_) Surveillance camera recording. (**a**_II_) Reception desk and (**a**_III_) electrode array position. (**b**) ICA maps. Red color indicates highest muscle activation. Wireless training of closing the eyes, wrinkling the nose and smiling voluntarily tasks revealed the three principle ICs: the *orbicularis oculi*, the *levator labii superioris* and the *zygomaticus major* muscles. (**c**,**d**) Real-time recordings of a 20 s conversation with a salesman. IC maps were calculated for segments I (1.3 s), II (3 s), VII (1.8 s) and VIII (1 s) indicating complex facial expressions involving the muscles mentioned above.

Having established the mapping of the *orbicularis oculi*, the *zygomaticus major*, and the *levator labii superioris* muscles, we can now turn to demonstrate the activation of these muscles in a natural setting. Our purpose was to show that a wireless version of the recording system could provide results similar to those detailed above. We used the reception desk at Tel Aviv University Center for Nanoscience and Nanotechnology (without introducing any modifications) as an experimental setup. The receptionist agreed to wear an electrode array with a wireless amplifier for several hours (7:00 a.m. to 1:00 p.m.) (Fig. 5a). We used a wireless amplifier system based on Intan RHD2000 amplifiers and Bluetooth Low Energy (BLE) V4.2 protocol for continuous data transfer to an Android smartphone^26^.

Data were recorded while the receptionist performed her regular duties, including answering the phone, processing purchase orders, and interacting with personnel and visitors. A surveillance camera (used routinely) recorded the activity (Fig. 5a). At first, the receptionist was asked to perform the three training tasks: closing the eyes, wrinkling the nose and smiling voluntarily. Each task was performed 5 times with 3 s neutral expression in between. The fastICA algorithm was applied to each repetition separately for the three tasks. Using the methodology described above, we identified the primary ${\hat{\theta }}_{i}$ due to consistency in muscle activation over all consecutive repetitions of the three training tasks: ${\hat{\theta }}_{{\rm{I}}}^{cEyes}=0.89\pm 0.01$ was identified as the *orbicularis oculi*; ${\hat{\theta }}_{{\rm{I}}}^{wNose}=1.12\pm 0.01$ as the *levator labii superioris*; and ${\hat{\theta }}_{{\rm{II}}}^{vSmile}=0.81\pm 0.02$ as the *zygomaticus major* muscle (Fig. 5b). Figure 5d shows a 20 s differential sEMG data segment during a conversation with a salesman standing next to the reception desk (recorded at 10:22 a.m.) (Fig. 5a_I_). Segments I-VII in Fig. 5c,d, demonstrate clear facial sEMG activations varying from 1 to 3 s. The fastICA algorithm was applied to these segments separately. The real-life IC maps reveal complex facial expressions ranging from activation of the *levator labii superioris* alone (Figure c_I_), a Duchenne-smile (combining the *zygomaticus major* and *orbicularis oculi* muscles together) (Figure c_II_), a non-Duchenne smile (activating only the *zygomaticus major* muscle) (Figure c_VII_) and finally, a complex-masked smile activating the three muscles together (Figure c_VIII_) (this classification relies on previous studies differentiating between smile types^5,6,9,13^). The participating components were identified by the ICs relative angles, ${\hat{\theta }}_{i}$.

The receptionist was first witnessed genuinely laughing and later smiling. When she was asked about her subjective impression of the encountered social interaction, she described it as “persistent” and “nagging”. This emotional description is in line with the observed IC maps suggesting the recognition of three different smile types^5^, in particular the “masked” smile. To the best of our knowledge, these are the first sEMG recordings of Duchenne (Fig. 5c,d_II_), non-Duchenne (Fig. 5c,d_VII_) and a complex-masked (Fig. 5c,d_VIII_) smiles in a natural face-to-face interaction.

## Discussion

In this study, we describe a high resolution, non-invasive sEMG method for objective mapping of facial muscle activation in both a lab and natural environment. A major drawback of contemporary facial sEMG is the interference of the experimental setups with the subject’s attention. Once the electrodes are placed on the face (ideally in a surreptitious manner), the attention of the subject should not be interrupted by any part of the experimental setup^8^. The soft and dry electrode arrays demonstrated here were optimized to achieve such an interface, allowing a free and natural behavior without the need for visual monitoring of the face. A wireless version of the system in a real-life environment enabled the identification of specific muscles commonly associated with “enjoyment”, “social” and “masked” smiles in an un-staged interaction. We note that the experiment reported here did not systematically (N = 1) validate the emotional state of the subject to allow association between facial muscle activation and emotion expression. Such examination would require methodic psychological evaluation, which is beyond the scope of the current report. Although demonstrated in a single subject and relying on her self-reporting, we provide proof of concept of facial sEMG recordings and analysis in a daily life environment with an unmodified setting.

A second fundamental challenge we addressed in this work is resolution, and in particular specific muscle (source) identification. Previous sEMG studies relied on exact electrode placement and provided statistical data^20,27^. Here we mapped the sources in a subject-specific manner, insensitive to electrode precise placement and anatomical diversity. Using a short training procedure and an ICA adapted methodology, we mapped the *orbicularis oculi*, the *levator labii superioris*, and the *zygomaticus major* muscle regions. Moreover, the ICs relative angles enabled the comparison and identification of the corresponding muscles between individuals. Overcoming the placement challenge implies that sEMG can be used in an automated manner, which is critical for quick analysis and evaluation in real-life applications.

An additional main challenge we addressed in this investigation is the complex muscle activation and interpersonal diversity patterns exhibited by different individuals. This challenge was resolved by noting that some activations are robust (over repetitions and individuals) and can be identified by the relative angle, a simple yet useful tool which we demonstrated above.

The ICA algorithm used and validated in this study achieved a clear separation between different facial muscles for the three muscles studied in all subjects and tasks. Yet, it was limited when the sEMG RMS was similar to the baseline noise RMS (SNR ≈ 1) or when the number of muscles activated exceeded the electrode number. The ICA mapping was projected to the area of the electrode array, and accordingly did not account for muscle activation beyond the contour of the array. In the present study, interpolation alone was used, however extrapolation may be tested in future work to address the challenge of distant muscle recordings. The 8 electrode configuration used in this study was sufficient to discriminate between the 3 regions of interest. It is likely that muscle identification can be further improved by increasing the electrode number, density, and targeting different areas of interest (such as the upper half of the face). A high density array positioned at the vicinity of diverse muscle regions, combining specific discriminating tasks, may allow better muscle distinction. Increasing the electrode number is straightforward engineering, involving the introduction of a higher density zero-insertion-force (ZIF) connector. An increased electrode number will also allow better muscle mapping, avoiding muscle identification at array edges, as well as the use of pattern recognition tools to identify IC similarity (beyond the simple relative angle comparison demonstrated here). For example, the histogram of the *zygomaticus major* muscle activation showed pronounced scattering compared with the other muscles. Better resolution and IC identification methodology may resolve this issue. Moreover, in this investigation, data analysis still relied on manual examination and will have to be further automized to render the system truly convenient for use.

In this investigation, we focused on muscles which had strong activation and were consistently activated in each facial expression in all subjects. The identification of these muscles is therefore robust. The origin of the additional components we observed is unclear and may be a result of poor SNR and a limitation of the fastICA algorithm. We cannot exclude that the unknown components resulted from the convergence of the algorithm. As the number of muscles activated in each facial expression is a-priori unknown, we did not limit the number of extracted output components in the fastICA. The convergence of each component was limited by 1000 steps, where in practice, tens of steps were sufficient. The physiological significance of the unknown components remains vague, but could to be investigated by artificially restricting the number of output components.

Primary facial muscles employed in speech include *mentalis, depressor anguli oris, masseter, digastricus, zygomaticus major, and orbicularis oris*^28,29^. Thus, even though the electrode array was customized a priori for the detection of smiles, speech could have been detected in the natural setting. To answer this question, an additional side experiment was performed to record motion and speech artifacts with the wireless system (Supplementary Fig. S3). Typical single-ended sEMG associated with speech and motion artifacts had low SNR values and therefore posed limited interference factor to the experiments. Moreover, as movement artifacts in the tattoo-like electrodes are similar for all electrodes, differential (filtered) data was almost movement artifact free. Another drawback of the wireless system was the sampling rate, limited by the capability and performance of the hardware, to meet the Bluetooth connectivity specifications. Theoretically, this may have influenced the capability of the fastICA to identify the sources. However, the main energy of the EMG signal is found between tens of Hz to below 200 Hz^30,31^, which is within the frequency spectrum of the recorded signals.

Facial expressions require the coordination of many muscles, activated synergistically, forming diverse actions in specific orders, both temporally and spatially^32,33^. Thus, the distinction of one facial expression versus another may involve not only the action of specific muscles, but also dynamic properties and their synergies. In this case, IC maps may correspond to a mixture of several muscles activated together rather than a single one. Beyond synergy of muscles, bilateral activation of the face may also be relevant. In the analysis above we used data from one cheek, although for most volunteers data from two cheeks are available. Analyzing the effects of asymmetry can further enhance our analysis and will be the scope of future investigation.

Finally, although our study focused on technical aspects, two interesting physiological results emerged. First, sEMG data during the closing the eyes task shows consistent activation synergy between the *orbicularis oculi* and the *levator labii superioris* muscles, which was revealed by identifying the primary ICs in both closing the eyes and wrinkling the nose tasks. This finding may be of relevance for research and treatment for pathological conditions of face muscles^34^. Second, in demonstrating the ability to measure “masked” smiles, our results suggest that sEMG of smiles may be an important tool to monitor human interaction in social real-life environments. Past sEMG investigations focusing on differentiating between positive and negative emotions typically targeted the activation of the *zygomaticus major* and *corrugator supercilii* muscles (smiling and frowning, respectively)^2,35^. However, in real face-to-face interactions, masked expressions may be socially more appropriate than the expression of outright negative emotions and therefore of greater relevance. In the current investigation, we studied a small number of expressions. Future studies involving additional muscles of the face and a wider emotional repertoire can contribute to better characterization of facial expression. Furthermore, our methodology can be extended to both general and specific body sEMG investigations.

To conclude, the technology outlined here established a novel perspective to sEMG that is user friendly and is indifferent to facial feature variance, user-expertise, and accurate electrode localization. As such, this facial sEMG system offers superior high-resolution performances with a crosstalk-free identification of specific muscles and excellent user convenience. This opens up new opportunities in gaming, virtual-reality, marketing, bio-feedback applications and objective psychological and neurological evaluation. Clearly, the methodology we presented above can be readily applied to many other muscle systems with numerous applications in diagnostics and rehabilitation.

## Methods

### Experimental setup and preprocessing

Eighteen healthy volunteers participated in the described facial movements’ experiment. Fifteen agreed to be laterally photographed posing a natural expression (used for ICA analysis). Each subject performed three types of movements (closing the eyes forcefully, wrinkling the nose and voluntary smiles) with 9 repetitions each (subject YK8014 and DS8017 did not perform the nose-wrinkling task properly (as evaluated by video recordings), closing the eyes data of subject YK8015 was excluded due to electrical artifacts. All subjects were thoroughly informed about the sEMG examination and gave written consent to participate in the study. The study was approved by the Tel Aviv University Ethics Committee (24/05).

A customized array of 8 electrodes (5 mm in diameter) was adhered to the subjects’ cheeks. The exact location of each electrode (relative to the origin (0,0) at electrode 0) is detailed in Supplementary Fig. S4. The electrode array design was attached to 5 different individuals prior to manufacturing the screen printing mask for proportion adjustments. The electrodes were screen printed using a conductive carbon ink as was previously described in^19^. The skin was mildly cleaned and exfoliated (everi, Spesmedica) prior to electrode placement.

sEMG data was recorded with sampling rate of 3000 (for the wired) and 410 (wireless) Samples/s. Data was filtered using a notch filter at 50 Hz and a band-pass 4 order Butterworth filter in the frequency range of 5–500 Hz (Wired) and 5–204 Hz (Wireless). Baseline noise RMS levels were calculated during the muscle’s relaxation time (neutral expression). SNR was calculated by dividing sEMG signal RMS levels (calculated over a period of activation) by the baseline noise RMS (5000 samples ≈ 1.67 s and 1000 sample ≈ 2.44 s for the wired and wireless systems, respectively). In both systems, the amplifier was attached to the subject cheek with a medical tape (3M, Transpore) and a plastic hair clip to assure adhesion and increase electrode-skin contact. The wireless system specifications were: weight = 7.95 gr; width = 3.5 cm; length = 1.8 cm and depth = 0.9 cm.

### FastICA algorithm

The fastICA ‘pow3’ nonlinearity function was used to calculate the ICs from the sEMG data. The fastICA was not limited in number of extracted output components and always resulted in 8 components. The convergence of each component was limited by 1000 steps. IC maps were interpolated to the lateral photographs resulting in 3264 × 2448 pixels resolution (1 pixel ≈ 0.08 mm). The JKLMN pentagon surface area was 869.75 mm^2^.

### Statistical analysis

Two linear mixed effect regression models were performed to predict the relative angle, ${\hat{\theta }}_{i}$. The first with a random Subject effect and a fixed IC effect. The second with a random Subject effect, fixed IC and Task effects and their interaction. Bonferroni correction was used for the multiple comparison. The statistical analysis was performed using R software 3.4.0.

### Experiments on Human Subjects

All experiments on human skin were conducted on volunteers in accordance with relevant guidelines and regulations under approval from the Institutional Ethics Committee Review Board at Tel Aviv University. Informed consent was obtained from all subjects.

### Data availability

The data that support the findings of this study are available upon request from the corresponding author (L.I.). The data are not publicly available due to ethical restrictions.

## Electronic supplementary material

Supplementary Information
