# Supplementary material for: A Wearable High-Resolution Facial Electromyography for Long Term Recordings in Freely Behaving Humans
Source: Sci Rep. 2018 Feb 1;8:2058. doi: 10.1038/s41598-018-20567-y (PMC5794977; doi:10.1038/s41598-018-20567-y)
Supplement: Supplementary file 1 — Supplementary Information [file 41598_2018_20567_MOESM1_ESM.docx]

**Supplementary Information**

**A Wearable High-Resolution Facial Electromyography for Long Term Recordings in Freely Behaving Humans**

Lilah Inzelberg^1,3,*^, David Rand^1^, Stanislav Steinberg^2^, Moshe David-Pur^1^, and Yael Hanein^1,2,3^

^1^Tel Aviv University Center for Nanoscience and Nanotechnology, Tel Aviv University, Israel

^2^School of Electrical Engineering, Tel Aviv University, Israel

^3^Sagol School of Neuroscience, Tel Aviv University, Israel

* e-mail: [lilahinz@mail.tau.ac.il](mailto:lilahinz@mail.tau.ac.il)

# **Supplementary Results**

The electrode array outlined in this study was designed to capture muscle activation at the lower half of the face and thus did not capture distant muscle activation such as the *corrugator supercilii*. To validate this, a subject was instructed to perform four facial movement tasks, namely: closing the eyes, wrinkling the nose, smiling voluntarily (taken from the original experiment) and wrinkling the eyebrows (typically activating the *corrugator supercilii* region ^1^). Supplementary Fig. S2 shows the filtered sEMG single-ended data of the four tasks: smiling voluntarily was recorded in all electrodes ($SNR = 2.65\pm0.99$), closing the eyes was mainly picked by electrode 6 and 7 ($SNR = 2.07\pm0.31$), wrinkling the nose mainly activated electrodes 2 and 4 ($SNR = 1.21\pm0.07$) and wrinkling the eyebrows was not recorded in any of the electrodes ($SNR = 1.06\pm0.01$).

Wireless facial sEMG recordings in an unmodified environment is a great challenge mainly due to movement and speech artifacts. To examine noise interference, a systematic experiment, introducing motion artifacts was performed. The same electrode array used in this investigation was positioned on the right cheek of a subject. The subject was instructed to perform diverse tasks presented on a computer monitor: smiling voluntarily (from the original imitation task), ‘Talk about the weather’, ‘Talk about your day’, ‘Move your head’, ‘Move your hands’, ‘Stand up’, ‘Walk in the room’ and ‘Sit down’. An additional video of a baby smiling was shown. When a black screen was presented (3 s), the subject was asked to remain with a neutral expression. While the raw single-ended data depicts some of the motion artifact noise, the filter (specified in the Methods) reduces most of the low frequency noise. Moreover, as movement artifacts in the tattoo-like electrodes are the similar for all electrodes, differential (filtered) data is almost movement artifact free (Supplementary Fig. S3). Single-ended sEMG of smiling voluntarily and smiling spontaneously (in response to the video) showed high amplitudes in all electrodes with SNR of $4.44\pm2.70$ (differential $SNR = 9.00\pm2.61$) and $2.67\pm0.86$ (differential $SNR = 4.96\pm2.15$), respectively. For speech and movement artifacts the SNR values were $1.4\pm0.19$(differential $SNR=1.29\pm0.39$) and $1.59\pm0.17$ (differential $SNR =1.50\pm0.32$), respectively.


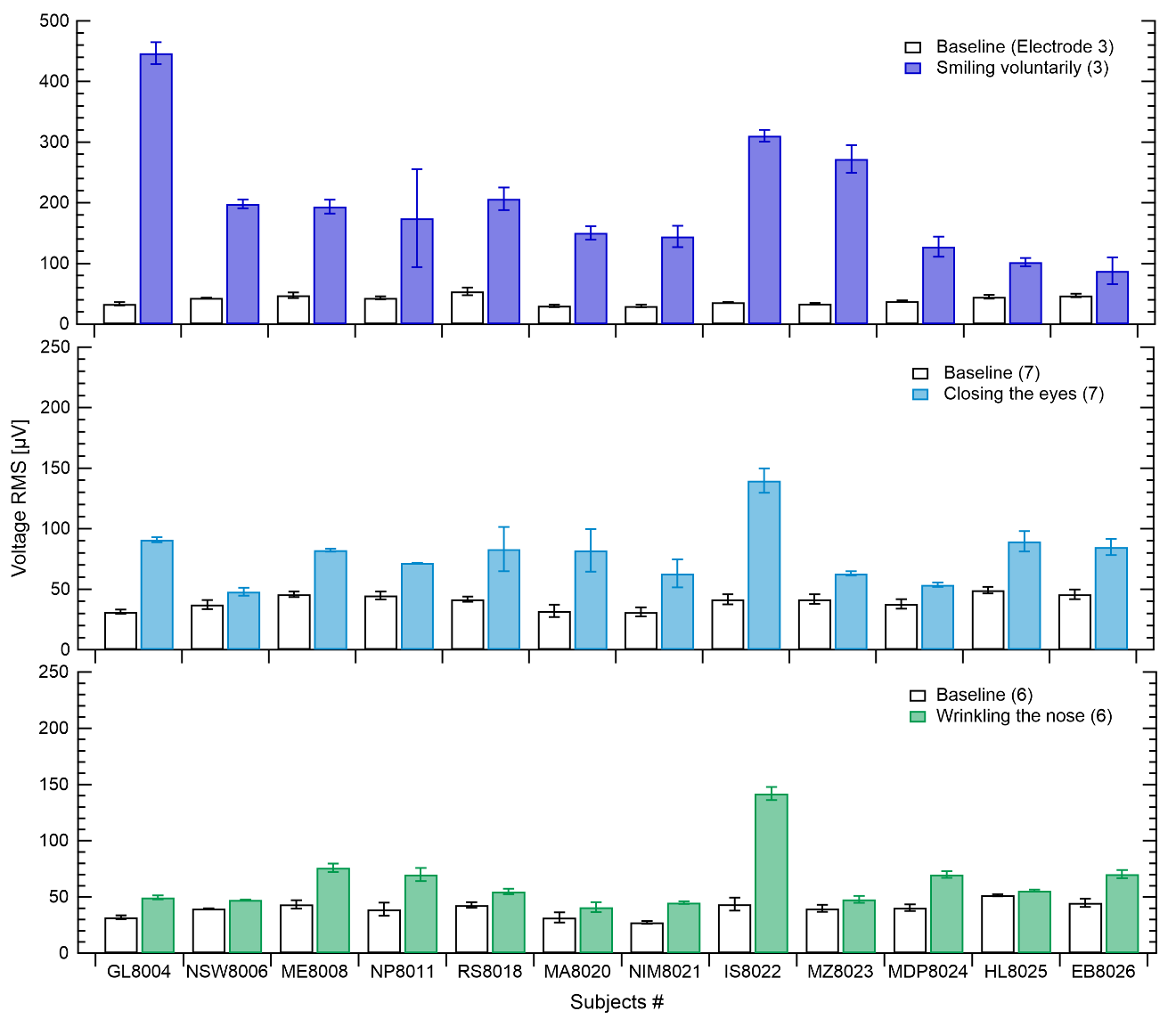


Supplementary Figure S1. Mean sEMG signal and baseline noise values (over 3 repetitions) for 12 subjects performing three facial movement tasks . (a) Closing the eyes (electrode 7); (b) wrinkling the nose (electrode 6) and (c) smiling voluntarily (electrode 3). Baseline noise RMS levels were always lower than that of the activation periods, over all electrodes and subjects.


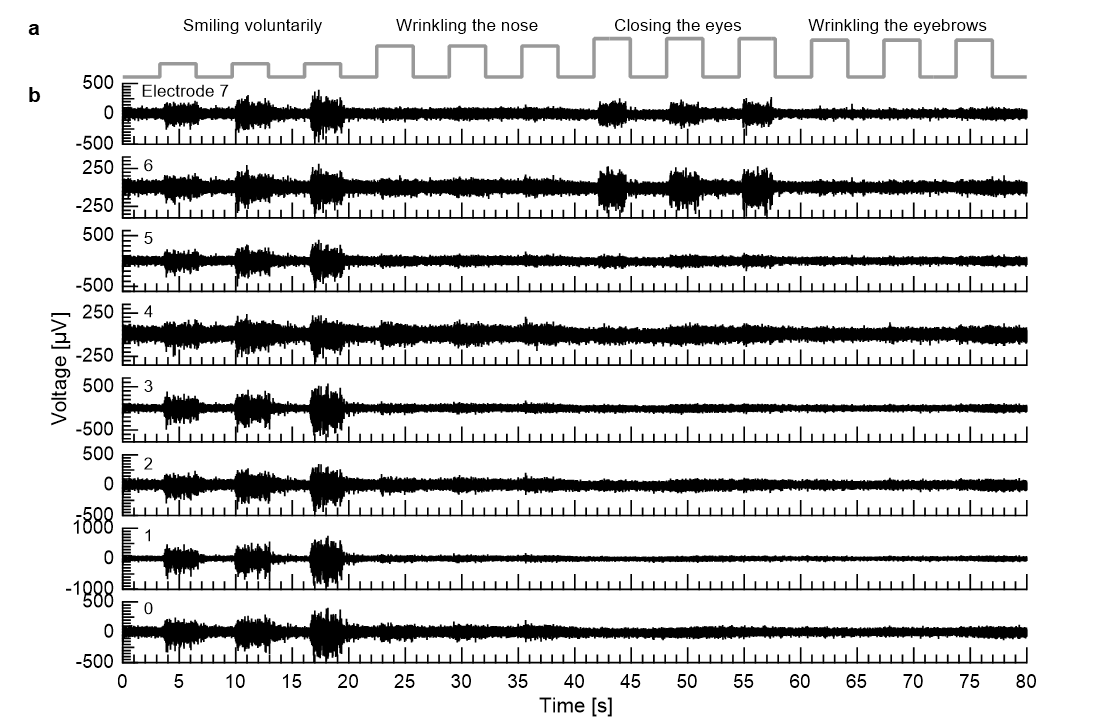


Supplementary Figure S2. Four facial voluntary activations and their corresponding 8 sEMG single-ended data (filtered) recorded from a single subject. (a) Task sequence shown to the subject: smiling voluntarily, wrinkling the nose, closing the eyes (taken from the original experiment) and wrinkling the eyebrows. (b) Single-ended sEMG recordings (voltage versus time). Smiling voluntarily typified by a large amplitude activation in all electrodes; wrinkling the nose was observed mainly in electrodes 2 and 4, closing the eyes was picked by electrode 6 and 7 and wrinkling the eyebrows was not recorded in any of the electrodes (baseline noise RMS was similar to the sEMG RMS).


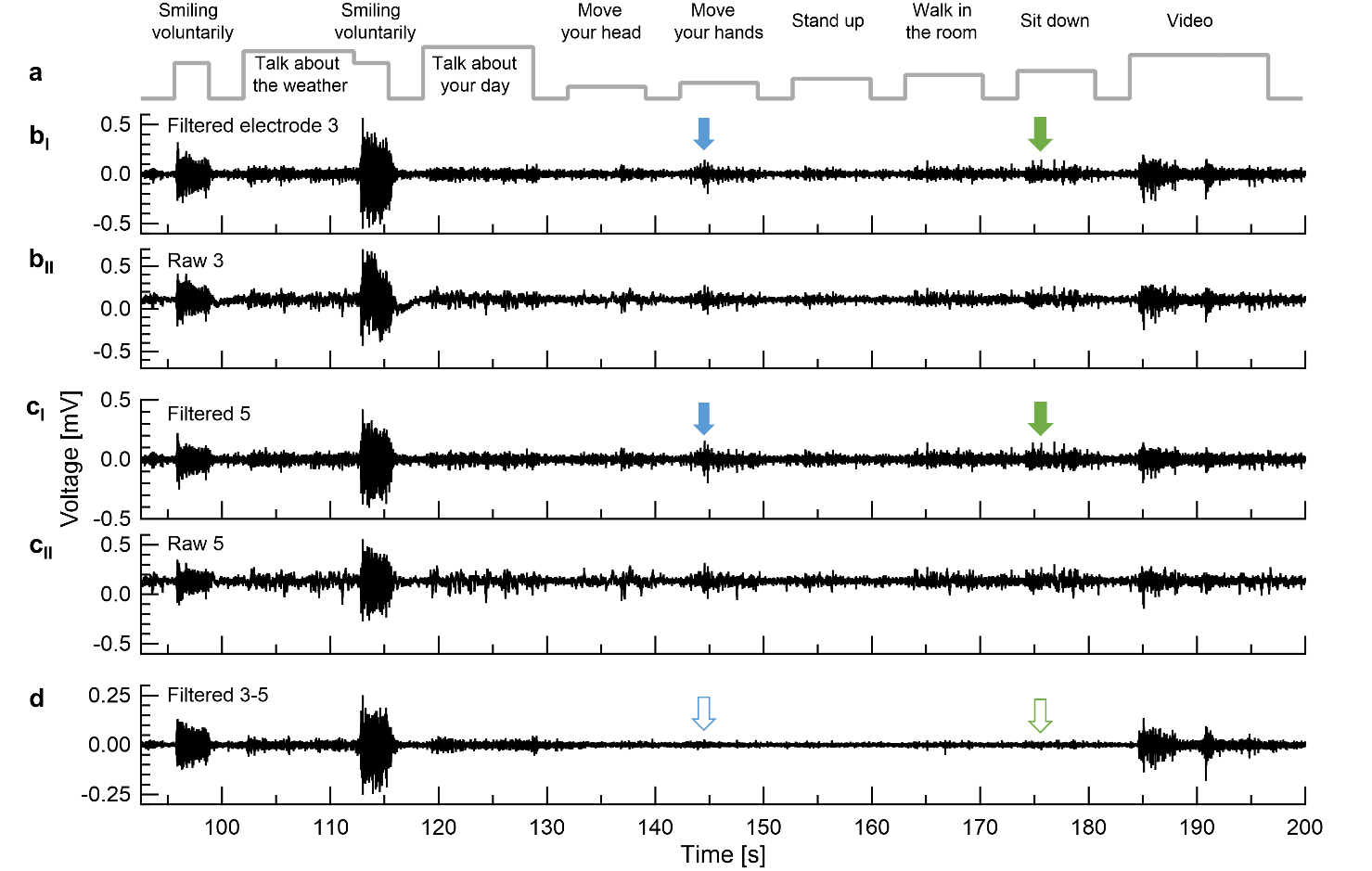


Supplementary Figure S3. sEMG data (voltage versus time) from a single subject performing different tasks aimed to introduce motion interference: ‘Talk about the weather’ (10 s), ‘Talk about your day’ (10 s), ‘Move your head’ (7 s), ‘Move your hands’ (7s), ‘Stand up’ (7 s), ‘Walk in the room’ (7 s) and ‘Sit down’ (7 s). Smiling voluntarily (3 s) and watching a video of a baby smiling (13 s) was also performed by the subject. (a) Task sequence shown to the subject. (b_I-II_) Raw single-ended and filtered sEMG signals recorded from electrode 3. (c_I-II_) Raw single-ended and filtered sEMG signals recorded from electrode 5. (d) Differential filtered sEMG signal (electrode 3 minus electrode 5). Smiling voluntarily and spontaneously (in response to the video) revealed high amplitude sEMG in all electrodes. Speech and movement artifacts were reduced by the filter and the differential presentation. Filled blue and green arrows highlight movement artifacts. Hollow arrows demonstrate motion artifact cancelation in the differential data.


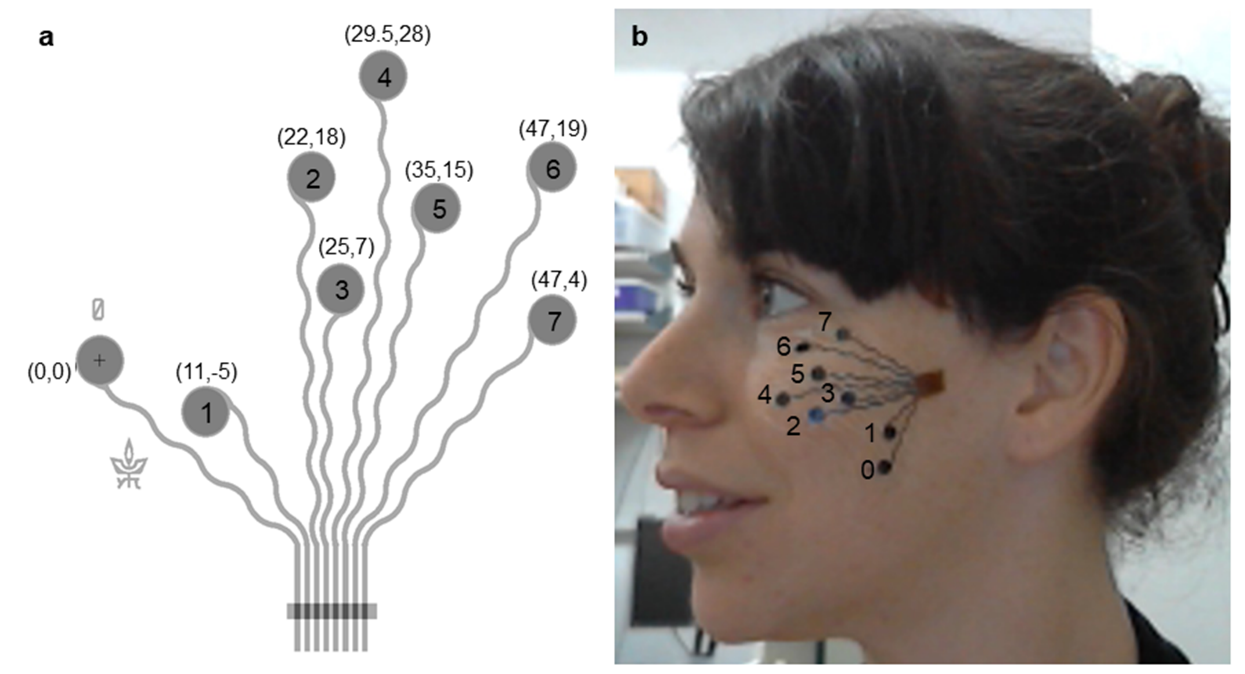
Supplementary Figure S4. The designed 8 electrode array. (a) Electrode positions [mm]. The origin (0,0) is set to the center of electrode 0. (b) An electrode array attached to a subject’s cheek.

**References**

1. Schumann, N. P., Bongers, K., Guntinas-Lichius, O. & Scholle, H. C. Facial muscle activation patterns in healthy male humans: A multi-channel surface EMG study. *J. Neurosci. Methods* **187,** 120–128 (2010).
